# A Multivariable Model of Parent Satisfaction, Pain, and Opioid Administration in a Pediatric Emergency Department

**DOI:** 10.5811/westjem.2021.6.51054

**Published:** 2021-09-02

**Authors:** Candice D. Donaldson, Theodore W. Heyming, Louis Ehwerhemuepha, Brooke N. Jenkins, Michelle A. Fortier, William Feaster, Zeev N. Kain

**Affiliations:** * Chapman University, Department of Psychology, Orange, California; † University of California, Irvine, Center on Stress & Health, Orange, California; ‡ Children’s Hospital of Orange County, Orange, California; § University of California, Irvine, Department of Anesthesiology and Perioperative Care, Orange, California; ¶ University of California, Irvine, Sue & Bill Gross School of Nursing, Irvine, California; || Yale Child Study Center, Yale University, New Haven, Connecticut

## Abstract

**Introduction:**

Children and adolescents are not impervious to the unprecedented epidemic of opioid misuse in the United States. In 2016 more than 88,000 adolescents between the ages of 12–17 reported misusing opioid medication, and evidence suggests that there has been a rise in opioid-related mortality for pediatric patients. A major source of prescribed opioids for the treatment of pain is the emergency department (ED). The current study sought to assess the complex relationship between opioid administration, pain severity, and parent satisfaction with children’s care in a pediatric ED.

**Methods:**

We examined data from a tertiary pediatric care facility. A health survey questionnaire was administered after ED discharge to capture the outcome of parental likelihood of providing a positive facility rating. We abstracted patient demographic, clinical, and top diagnostic information using electronic health records. Data were merged and multivariable models were constructed.

**Results:**

We collected data from 15,895 pediatric patients between the ages of 0–17 years (mean = 6.69; standard deviation = 5.19) and their parents. Approximately 786 (4.94%) patients were administered an opioid; 8212 (51.70%) were administered a non-opioid analgesic; and 3966 (24.95%) expressed clinically significant pain (pain score >/= 4). Results of a multivariable regression analysis from these pediatric patients revealed a three-way interaction of age, pain severity, and opioid administration (odds ratio 1.022, 95% confidence interval, 1.006, 1.038, P = 0.007). Our findings suggest that opioid administration negatively impacted parent satisfaction of older adolescent patients in milder pain who were administered an opioid analgesic, but positively influenced the satisfaction scores of parents of younger children who were administered opioids. When pain levels were severe, the relationship between age and patient experience was not statistically significant.

**Conclusion:**

This investigation highlights the complexity of the relationship between opioid administration, pain severity, and satisfaction, and suggests that the impact of opioid administration on parent satisfaction is a function of the age of the child.

## INTRODUCTION

### Background

The United States is currently experiencing an unprecedented opioid crisis. More than 47,000 people die from an opioid-related overdose each year,^1^ and the annual economic burden is estimated to be $504 billion.^2^ Children and adolescents are not impervious to this epidemic. In 2016 alone, more than 88,000 adolescents between the ages of 12–17 years reported misusing opioid medications, making opioids the second most commonly abused illicit substance in the US.^3^ Physician prescribing is argued to be one driver of the opioid crisis.^4^ One major source of prescribed opioids is the emergency department (ED),^5^ with the treatment of pain identified as the most common reason for ED visits.^6^,^7^ Specifically, guidelines^8^ focused on improving the pain management of pediatric patients and reducing the undertreatment of pain in children with a variety of painful conditions^9^ may have influenced the increase in opioid prescriptions to children and adolescents.

### Importance

With a rising emphasis on patient-centered care, concerns over patient satisfaction may be one contributor to the increased opioid prescribing rates observed in the ED.^10^ Patient satisfaction is an important tool for assessing quality of care, and with the widespread availability of several commercially available surveys that capture patient experience, results of these questionnaires can now impact a facility’s reputation and profits. As a result, physicians may fear that insufficiently treating pain could lead to decreased patient satisfaction, which would contribute to the continued opioid prescribing habits of these providers.^11^ Research on the relationship between patient satisfaction and pain management in adult populations is mixed. Some studies have determined that analgesic administration does not correlate with patient satisfaction,^12^,^13^ whereas, a significant link between pain management and quality of care has been shown in other investigations.^14^–^16^

Studies examining this correlation in pediatric samples have received little attention,^17^,^18^ and there is an absence of research that addresses potential statistical interactions between pain severity and the administration of opioids in relation to patient satisfaction in one multivariable model. Margaret and associates found that pain resolution was associated with higher satisfaction; however, differences based on analgesic administration were not assessed.^18^ Similarly, Locke and colleagues showed that patients indicating their pain was controlled were more satisfied with their ED experience. But analgesic use was not examined in this investigation.^17^ Finally, given the great developmental differences among children ages 0–17 it is necessary to assess how the association between pain and opioid prescribing impacts satisfaction at different age levels.

### Goals of This Investigation

The relationship between opioid administration, pain severity, and parent satisfaction (as a proxy of patient satisfaction) is complex and should be assessed using a multivariable analysis that simultaneously considers the impact of both analgesic administration and pain on parent satisfaction. Research that assesses only the role of either pain or opioid use, without including both, will fail to fully capture the complex contribution of opioid administration on perceived quality of care. Therefore, our goal was to determine the influence of opioid analgesic administration on parent satisfaction for pediatric patients discharged from the ED, and to assess whether the pain management-satisfaction relationship was impacted by demographic, clinical, and diagnostic factors.

**Population Health Research Capsule**

What do we already know about this issue?

*In adults, research has identified a significant association between pain management and patient satisfaction; however, this relationship is understudied in pediatric populations*.

What was the research question?

*We assessed statistical interactions between age, pain, and the prescription of opioids in relation to parental satisfaction with care*.

What was the major finding of the study?

*Parents of older patients were dissatisfied with their care when their child was prescribed opioids to treat a milder pain condition*.

How does this improve population health?

*Findings suggest that physicians should consider pediatric patient pain level and age when deciding whether to prescribe an opioid medication*.

## METHODS

### Study Design and Setting

The current study involved a retrospective cohort analysis of parent satisfaction with analgesia administration in the ED. We collected encounter data between May 2018–June 2019 from children who underwent treatment in a pediatric ED in a tertiary children’s hospital. This data source included demographic and clinical variables from an electronic health record (EHR) system and parent satisfaction data that we assessed using a NRC Health survey questionnaire (National Research Corporation, Markham, Ontario, Canada). In total, the parents of 85,804 ED patients began to answer survey questions, and of these 24,761 respondents completed the survey, representing a response rate of 28.9%. All survey data collection methods were approved by the hospital’s institutional review board.

### Selection of Participants

The health questionnaire was sent to all parents after discharge from the ED facility. We linked the EHR data and survey responses using unique encounter identifiers present in both data sources. Inclusion criteria were as follows: treated in the ED; being < 18 years; and with an ED stay of 12 hours or less. Because we aimed to assess differences in opioid prescribing for patients without cancer-associated chronic pain or a neoplasm diagnosis we excluded from the analysis International Classification of Diseases, 9^th^ and 10^th^ revisions, (ICD-9 and ICD-10) codes C00 through D49; n = 59).^19^,^20^

### Measurements

Patient age, ethnicity, gender, low-income insurance status (Medicare/Medi-Cal),* Emergency Severity Index score, length of stay (ranging from 16–716 minutes), and level of pain were abstracted from the EHR. Pain severity ranged from 0 (*no pain*) to 10 (*severe pain*), and was conceptualized as the maximum pain score recorded during the patient’s stay using developmentally and situationally appropriate measurement tools (ie, the Neonatal Pain, Agitation and Sedation scale; the Faces, Legs, Activity, Cry, Consolability behavioral pain scale; the faces pain scale, and numeric rating scale). Following the guidelines of Fortier et al,^21^ a pain score equal to or greater than 4 was determined to be clinically significant.

Patient analgesic records during the ED visit were also obtained from the EHR. Specifically, we assessed information on whether the patient was administered an opioid (eg, codeine, hydrocodone, hydromorphone, meperidine, sufentanil, fentanyl, morphine, oxycodone, remifentanil, nalbuphine, methadone, tramadol) and/or non-opioid analgesic (eg, ibuprofen, acetaminophen, naproxen, gabapentin, pregabalin, celecoxib, and triptan). Administered opioids were dispensed during the patient’s ED stay and did not refer to after-visit administrations, as information on medications prescribed after the patient’s ED stay was not accessible in the EHR system. Top patient medical diagnoses were also retrieved and controlled for using the ICD-9 and ICD-10 revisions (0 = *absence of a diagnosis*, 1 = *presence of a diagnosis*). Diagnoses that captured less than 1% of patients (eg, sickle cell disease) were not controlled for in the analyses. We used responses to the health questionnaire after patient discharge to measure patient experience. Two items assessing satisfaction with pain and discomfort management were examined (ie, “Did the staff do everything they could to help your child with his/her discomfort?”; and “Did the care providers do everything they could to ease your child’s discomfort?”).

### Outcome

We determined parent satisfaction with their child’s emergency care using an NRC Health questionnaire item assessed as an indicator of patient satisfaction in prior research.^22^ Specifically, parents were asked, “Using a number from 0 to 10, where 0 is the worst facility possible and 10 is the best facility possible, how would you rate this emergency department?” A top-box approach was used to recode this item, with a response of “9” or “10” indicating satisfaction with the ED facility (coded as 1 or “*Satisfied*”). All other responses represented an undesirable facility rating (coded as 0 or “*Not Satisfied*”). This top-box methodology is the standard approach for assessing patient satisfaction in US hospitals that use the Hospital Consumer Assessment of Healthcare Providers and Systems.^23^–^28^

### Analytic Approach

Bivariate analyses estimated associations between satisfaction with admittance acuity, pain severity, administered analgesics, and the two items assessing pain management experiences. We examined the proportions of patients with positive (*Satisfied*) and negative (*Not Satisfied*) facility rating scores across the levels of each variable. Odds ratios (OR) and chi-square *P-*values were calculated for each association. In addition, we assessed relationships between the two pain and discomfort management survey questions and opioid administration using a chi-square test of association. *P*-values < 0.05 were determined to be statistically significant in the bivariate analyses.

A multivariable logistic regression model was then estimated to assess the relationship between each predictor and the outcome while controlling for all demographic, clinical, survey, and diagnosis variables. Two-way interactions between age, ethnicity, gender, insurance type, acuity score, length of stay, and pain with opioid use were simultaneously estimated. We also assessed three-way interactions between each covariate with pain and opioid use. We removed non-significant interaction terms before estimating the final model. A Bonferroni correction was applied to account for the estimation of two models, with *P* < 0.025 concluded to be statistically significant in the multivariable analyses.

## RESULTS

### Characteristics of the Study Subjects

Respondents were 15,895 pediatric patients and their parents. Overall, 11,995 (75.46%) patients provided a positive ED facility rating, meaning that they were satisfied with their visit (providing a score of a 9 or 10 when asked how they would rate the ED facility). Approximately 3966 (24.95%) expressed clinically significant pain (pain score >/= 4; as defined by Fortier et al^21^); 786 (4.94%) patients were administered an opioid; and 8212 (51.70%) were administered a non-opioid analgesic. Additional descriptive information is displayed in Table 1.

### Main Results

#### Bivariate Analyses

Relationships between all key variables with satisfaction are shown in Table 2. Patients with an acuity score of 1 or 2 (Resuscitation/Emergent) were more likely to provide a positive facility rating than patients with an acuity score of 3 (Urgent) or 4/5 (Less Urgent/Non-Urgent; *P* = 0.002). Patients expressing more severe pain severities (*P* < 0.001) and those who were administered opioid analgesics (*P* = 0.018) were more likely to report they were satisfied with the ED facility. Parents who indicated that the staff and care providers “definitely” did everything they could to help the child with their discomfort were most likely to be satisfied with their patient experience in the ED (both *P*-values < 0.001).

We also assessed associations between the two pain and discomfort management survey items and opioid administration. Parents of patients administered opioids were more likely to indicate that staff “definitely” did everything they could to help with the child’s discomfort (n = 563, 71.60%) than patients who were not given opioids (n = 9898, 65.50%; χ2 (4) = 14.54, *P* = 0.006). Similarly, compared to parents whose children did not receive an opioid (n = 6152, 40.70%), those whose child received an opioid analgesic were more likely to indicate that care providers “definitely” did everything they could to ease their child’s discomfort (n = 340, 43.30%, χ2 (4) = 11.52, *P* = 0.021).

#### Multivariable Analysis

A multivariable model (see Table 3) controlling for all demographic, clinical, and top diagnosis covariates showed that patients with an acuity score of 1 or 2 (Resuscitation/Emergent) were more likely to provide a positive ED evaluation than patients with an acuity score of 4/5 (Less Urgent/Non-Urgent; *P* < 0.001). Staying in the ED for a shorter time (ie, shorter length of stay, *P* < 0.001) and being administered an opioid analgesic (*P* = 0.007) were both associated with a greater likelihood of indicating a positive facility rating. Interestingly, the administration of non-opioid analgesics was not associated with parent satisfaction (*P* = 0.131). Also, parents who reported that staff and care providers “definitely” did everything they could to help the child manage their discomfort were more likely to be satisfied than parents that responded “Yes, mostly,” “Yes, somewhat,” or “No” (all *P* < 0.001).

**Figure 1:**
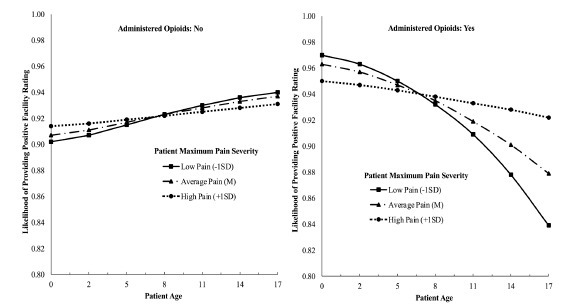
Three-way interaction of age, pain severity, and opioid administration on parent likelihood to provide a positive facility rating controlling for all other demographic, clinical, and top diagnosis covariates. Age by pain severity interactions were graphed for patients were not given an opioid (left) and for those that were administered an opioid (right).

We assessed the complex relationships between pain, opioid administration, and parent satisfaction by estimating several interaction terms. Findings revealed a significant three-way interaction of age, pain severity, and opioid administration (Figure 1, *P* = 0.007). To decompose the three-way interaction, simple slopes of the relationship between age and facility rating were estimated and graphed on the pain severity moderator at one standard deviation (SD) below the mean, at the mean, and one SD above the mean for patients who were and were not administered an opioid analgesic during their ED stay.^29^ All covariates were controlled for in this model. For patients *not administered* an opioid analgesic, there was no statistically significant relationship between age and facility rating when pain severity was high (+1 SD; *b* = 0.01, 95% confidence interval [CI], 0.00, 0.03, *p* = 0.08] or moderate (Mean; *b* = 0.03, 95% CI, 0.02, 0.04, *P* = 0.07). However, when pain was mild (−1 SD; *b* = 0.03, 95% CI, 0.02, 0.04, *P* < 0.001) and patients did *not* receive an opioid, parents of older patients reported greater satisfaction than parents of younger patients. Specifically, for patients who did not receive an opioid, parents of older patients, expressing mild pain, were the most satisfied with their experience in the ED.

For patients who were *administered opioids*, a different pattern emerged. The relationship between age and patient experience was not statistically significant when pain was more severe (+1 SD; *b* = −0.03, 95% CI, −0.07, 0.02, *P* = 0.26). Thus, for patients experiencing higher levels of pain, patient age did not impact parent satisfaction. However, when the level of pain was mild (−1 SD; *b* = −0.11, 95% CI, −0.19, −0.02, *P* = 0.01) or moderate (Mean; *b* = −0.07, 95% CI, −0.14, −0.01, *P* = 0.02) parents of younger children were more likely to provide a positive facility rating than parents of older patients. In other words, pain level did not strongly impact the parent satisfaction of the youngest patients. These patients were the most satisfied when their child received an opioid, regardless of pain severity. In contrast, pain level had an important influence on the parent satisfaction scores of the oldest patients who were given an opioid during their ED stay. The least satisfied group in this sample were parents of 17-year-old patients who were administered an opioid analgesic, despite expressing mild pain severity. For example, parents of the oldest patients (ie, 17-year-olds who were administered opioids were 9.43% more likely to provide a positive facility rating when their child was in more severe rather than mild pain (likelihood rating of 0.922 vs 0.839).

## DISCUSSION

Under the conditions of this study, results from the multivariable regression model revealed that parents of patients admitted with a resuscitation or emergent acuity score were more likely satisfied with the ED facility than patients who were admitted with a less urgent or non-urgent acuity rating. A multivariable analysis controlling for demographic, clinical, and top diagnostic covariates showed that opioid analgesic administration was related with parent satisfaction in a pediatric ED setting but non-opioid analgesics were not. Specifically, parents of patients administered an opioid analgesic were more likely to provide a positive facility rating compared to parents of patients who did not receive an opioid. Further, relationships between opioid administration and parent satisfaction were shown to be multifaceted and complex, as demonstrated by the significant interaction of age, pain, and opioid administration. That is, parents of younger patients who received an opioid were the most satisfied with their quality of care, regardless of pain severity; whereas likelihood to provide a positive facility rating substantially decreased for parents of the oldest patients who were administered an opioid to manage mild pain. Additionally, it is important to emphasize that diagnosis (eg, orthopedic injury) did not impact the pattern of results shown in this study, as the regression analyses controlled for common medical aliments.

With the current opioid epidemic, rates of pediatric opioid-related overdose and death continue to increase.^30^ Markedly, opioid prescribing has been identified as a risk factor of later misuse^31^ and persistent use^32^ in pediatric patients. Emergency medicine has been recognized as one of the top five specialties that prescribe prescription opioids,^5^ since most ED visits include the treatment of painful medical conditions.^6^,^7^ Consequently, concerns over patient satisfaction might be contributing to this epidemic,^11^ as previous studies have supported a common belief that administering opioid analgesics will improve patient experience scores in the ED.^14^–^16^ However, findings from this investigation indicate that the relationship between opioid administration and patient experience is more complex than originally believed, and depends on both the age of the patient and their level of pain. When older patients experienced severe pain and were treated with opioid analgesics, parents were satisfied with their ED experience; but, when these patients were given opioids when experiencing lower levels of pain, parents were unsatisfied with the care their child received.

The finding that opioid administration negatively impacted the patient satisfaction of parents with adolescent children admitted to the ED with lower levels of pain should be interpreted in the context of the opioid public health crisis. Studies with different samples of youth patients show that adolescence represents a transitional developmental period characterized by an increase in risky health behaviors, including illicit substance use experimentation.^33^,^34^ Given the link between prescribed opioid use and later misuse shown in other studies,^31^ it seems logical to conclude that parents in this study were dissatisfied with their child’s quality of care when given opioids for a milder pain condition, as exposing the patient to opioid medications in the face of milder pain could represent an unnecessary developmental risk.

This result implies that the administration of opioid medications does not always improve patient satisfaction, and that in some situations, it can actually make perceived clinical care worse. Thus, physicians should consider pain level and age when making decisions about whether to administer an opioid medication to pediatric patients. Also, from a methodological perspective, results of this study highlight the importance of examining pain, opioid use, and satisfaction simultaneously in one model, and imply that future studies should examine and control for all three variables to understand complex relationship between pain and discomfort management and patient experience.

## LIMITATIONS

Findings should be interpreted in the context of several limitations. Patients in this study were admitted to the ED of a single, tertiary pediatric institution; thus, findings might not be generalizable to all pediatric ED facilities. Parent satisfaction was measured via self-report. Therefore, parent responses might be impacted by acquiescence bias. Specifically, parents might have felt pressure to provide a positive ED rating although the survey was conducted after the parents left the ED. Information on opioid prescribing after discharge home could not be obtained, which might have served as a confounding variable potentially impacting parent satisfaction. The response rate in this study was 28.9%, which could have biased findings. However, research suggests a small association between response rates and nonresponse bias for surveys such as the NRC health questionnaire.^35^ Pain severity was recorded by care providers throughout the patient’s ED stay; thus, there could have been some error in the way that pain was interpreted and recorded. Additionally, timeliness of analgesia administration could not be captured in this study. Future investigations might assess changes in pain from triage to discharge to understand how pain management and medication administration impacts satisfaction.

## CONCLUSION

Findings from this investigation point to the multifarious nature of the relationship between pain severity, opioid administration, and parent satisfaction, and highlight a potential conflict in patient-physician interactions at the intersection of parent satisfaction and controlled substance administration. Multivariable analyses showed that parents of patients who were given opioids during their stay in the ED were more satisfied but that this relationship was also impacted by pain level and the age of the patient. Parents of older adolescent patients were dissatisfied with their ED experience when their child received opioids to treat a milder pain condition. Although encounters in the ED can be challenging due to time limitations and physician unfamiliarity with a patient’s background, our findings and the cited literature suggest that factors such as patient age, pain, acuity score, and possible risk of opioid misuse should be considered when administering opioid medications. Consequently, ED facilities might consider designing and implementing evidence-based policies and tools that help physicians quickly determine whether opioids should be administered for pain management based on the patient’s characteristics and unique risk of misuse.

## Figures and Tables

**Table 1:** Pediatric patient sample characteristics (N = 15,895).

Demographic and Clincal Predictors	
Age	
Range	0 – 17 years
M (SD)	6.69 (5.19)
Ethnicity	
Hispanic	5,786 (63.60%)
Non-Hispanic	10,109 (36.40%)
Sex	
Male	8,494 (53.44%)
Female	7,401 (46.56%)
Low-income insurance (medicare/medi-cal)	
No	4,419 (27.80%)
Yes	11,476 (72.20%)
Acuity Score	
1/2 (Resuscitation/Emergent)	1,192 (7.50%)
3 (Urgent)	5,516 (34.70%)
4/5 (Less Urgent/Non-Urgent)	9,187 (57.80%)
Length of stay (minutes)	
Range	16 – 716
M (SD)	170.00 (94.85)
Pain severity	
Range	0 – 10
M (SD)	1.86 (2.72)
Administered opioids	
No	15,109 (95.06%)
Yes	786 (4.94%)
Administered non-opioids	
No	7,683 (48.30%)
Yes	8,212 (51.70%)

Top International Classification of Diseases, Ninth/Tenth Revision Diagnoses	

Bacterial/viral infections (A00–A99)	1,149 (7.23%)
Circulatory system diseases (I00–I99)	159 (1.00%)
Congenital malformations, deformations and chromosomal abnormalities (Q00–Q99)	219 (1.38%)
Digestive and genitourinary system diseases (K00–K95, N00–N99)	1,764 (11.10%)
Ear and eye diseases (H00–H59, H60–H95)	1,351 (8.50%)
Endocrine, nutritional, and metabolic diseases (E00–E89)	253 (1.59%)
Mental and behavioral disorders (F01–F99)	556 (3.50%)
Diseases of the skin and subcutaneous tissue, musculoskeletal system, and connective tissue (L00–L99, M00–M99)	2,050 (12.90%)
Diseases of the nervous system (G00–G99)	283 (1.78%)
Diseases of the respiratory system (J00–J99)	2,956 (18.60%)
Single body region traumatic injuries non-orthopedic (S00–S391)	1,717 (10.80%)
Orthopedic injury (S40–S991)	2,273 (14.30%)
Unspecified body regions, poisonings, other consequences of external causes, and all other trauma or injury (T00–T141, T15–T791)	741 (4.66%)

*Note*. Only top medical diagnoses that captured >/= 1% of patients were assessed. *M*, Mean; *SD*, standard deviation.

**Table 2:** Results of bivariate analysis.

Variables	Levels	Not Satisfied n (%) or M (SD)	Satisfied n (%) or M (SD)	Odds Ratio (95% CI)	Chi-square P-value
Acuity Score	1/2 (Resuscitation/Emergent)	247 (20.83)	939 (79.17)	Reference	0.002*
	3 (Urgent)	1327 (24.07)	4187 (75.93)	0.83 (0.711, 0.966)	
	4/5 (Less Urgent/Non-Urgent)	2326 (25.30)	6869 (74.70)	0.777 (0.669, 0.899)	
Pain severity	-	1.73 (2.61)	1.91 (2.75)	1.026 (1.012, 1.04)	< 0.001*
Administered opioids	No	3735 (24.72)	11374 (75.28)	Reference	0.018*
	Yes	165 (20.99)	621 (79.01)	1.236 (1.04, 1.477)	
Administered non-opioids	No	1926 (25.07)	5757 (74.93)	Reference	0.131
	Yes	1974 (24.04)	6238 (75.96)	1.057 (0.983, 1.136)	
Did the staff do everything they could to help your child with his/her discomfort?	Yes, definitely	1274 (32.67)	9187 (76.59)	Reference	< 0.001*
	Yes, mostly	1023 (26.23)	1768 (14.74)	0.240 (0.218, 0.264)	
	Yes, somewhat	845 (21.67)	730 (6.09)	0.120 (0.107, 0.134)	
	No	710 (18.21)	146 (1.22)	0.029 (0.024, 0.034)	
	Not applicable	48 (1.23)	164 (1.37)	0.474 (0.345, 0.664)	
Did the care providers do everything they could to ease your child’s discomfort?	Yes, definitely	737 (18.90)	5755 (47.98)	Reference	< 0.001*
	Yes, mostly	607 (15.56)	1199 (10.00)	0.253 (0.223, 0.286)	
	Yes, somewhat	516 (13.23)	458 (3.82)	0.114 (0.098, 0.132)	
	No	455 (11.67)	94 (0.78)	0.026 (0.021, 0.033)	
	Not applicable	1585 (40.64)	4489 (37.42)	0.363 (0.329, 0.399)	

*Note*.

* denotes statistical significance at the p < 0.050 level.

**Table 3:** Results of multivariate analyses.

Main Effects	Odds Ratio (95% CI)	P-value
Age	1.034 (1.022, 1.046)	< 0.001*
---	1.034 (1.022, 1.046)	< 0.001*
Ethnicity		
Hispanic	Reference	
Non-Hispanic	0.644 (0.587, 0.706)	< 0.001*
Sex		
Female	Reference	
Male	0.9715 (0.892, 1.058)	0.507
Low-income insurance (medicare/medi-cal)		
No	Reference	
Yes	1.834 (1.660, 2.0260)	< 0.001*
Acuity Score		
1/2 (Resuscitation/Emergent)	Reference	
3 (Urgent)	0.545 (0.450, 0.658)	0.060
4/5 (Less Urgent/Non-Urgent)	0.545 (0.450, 0.658)	< 0.001*
Length of stay		
---	0.996 (0.996, 0.997)	< 0.001*
Pain severity		
---	1.034 (0.993, 1.076)	0.105
Administered opioids		
No	Reference	
Yes	3.537 (1.458, 9.089)	0.007*
Administered non-opioids		
No	Reference	
Yes	1.053 (0.963, 1.1535)	0.256
Did the staff do everything they could to help your child with his/her discomfort?		
Yes, definitely	Reference	
Yes, mostly	0.304 (0.272, 0.340)	< 0.001*
Yes, somewhat	0.177 (0.154, 0.203)	< 0.001*
No	0.058 (0.047, 0.071)	< 0.001*
Not applicable	0.614 (0.440, 0.872)	0.005*
Did the care providers do everything they could to ease your child’s discomfort?		
Yes, definitely	Reference	
Yes, mostly	0.515 (0.446, 0.596)	< 0.001*
Yes, somewhat	0.371 (0.310, 0.444)	< 0.001*
No	0.157 (0.120, 0.209)	< 0.001*
Not applicable	0.622 (0.559, 0.692)	< 0.001*

Interaction Effects	Odds Ratio (95% CI)	P-value

Age × opioid administration	0.869 (0.796, 0.947)	0.001*
Age × pain severity	0.996 (0.992, 0.999)	0.023*
Opioid administration × pain severity	0.859 (0.718, 1.0257)	0.095
Age × pain severity × opioid administration	1.022 (1.006, 1.038)	0.007*

*Note*. ICD 9/10 diagnoses were controlled for in the multivariate model but are not depicted in this table to maintain conceptual clarity.

* denotes statistical significance at the p < 0.025 level.
